# Molecular differences between younger versus older ER-positive and HER2-negative breast cancers

**DOI:** 10.1038/s41523-022-00492-0

**Published:** 2022-11-07

**Authors:** Tao Qing, Thomas Karn, Mariya Rozenblit, Julia Foldi, Michal Marczyk, Naing Lin Shan, Kim Blenman, Uwe Holtrich, Kevin Kalinsky, Funda Meric-Bernstam, Lajos Pusztai

**Affiliations:** 1 Breast Medical Oncology, School of Medicine, Yale University, New Haven, CT USA; 2 Department of Gynecology and Obstetrics, Goethe-University Frankfurt, Frankfurt, Germany; 3 Department of Data Science and Engineering, Silesian University of Technology, Gliwice, Poland; 4 Department of Hematology and Medical Oncology, Winship Cancer Institute, Emory University, Atlanta, GA USA; 5 Department of Investigational Cancer Therapeutics, The University of Texas MD Anderson Cancer Center, Houston, TX USA

**Keywords:** Prognostic markers, Cancer genomics

## Abstract

The RxPONDER and TAILORx trials demonstrated benefit from adjuvant chemotherapy in patients age ≤ 50 with node-positive breast cancer and Recurrence Score (RS) 0–26, and in node-negative disease with RS 16–25, respectively, but no benefit in older women with the same clinical features. We analyzed transcriptomic and genomic data of ER+/HER2− breast cancers with in silico RS < 26 from TCGA (*n* = 530), two microarray cohorts (A: *n* = 865; B: *n* = 609), the METABRIC (*n* = 867), and the SCAN-B (*n* = 1636) datasets. There was no difference in proliferation-related gene expression between age groups. Older patients had higher mutation burden and more frequent ESR1 copy number gain, but lower frequency of GATA3 mutations. Younger patients had higher rate of ESR1 copy number loss. In all datasets, younger patients had significantly lower mRNA expression of ESR1 and ER-associated genes, and higher expression of immune-related genes. The ER- and immune-related gene signatures showed negative correlation and defined three subpopulations in younger women: immune-high/ER-low, immune-intermediate/ER-intermediate, and immune-low/ER-intermediate. We hypothesize that in immune-high cancers, the cytotoxic effect of chemotherapy may drive the benefit, whereas in immune-low/ER-intermediate cancers chemotherapy induced ovarian suppression may play important role.

## Introduction

Most breast cancers are diagnosed in women older than 50^1^. Age is not only a risk factor for cancer, but it also interacts with adjuvant chemotherapy benefit in hormone receptor positive/human epidermal growth factor receptor-2 negative (HR+/HER2−) breast cancers^2^. Three randomized trials demonstrated greater chemotherapy benefit in younger compared to older women^3^. The TAILORx trial showed improved invasive disease-free survival (IDFS) with chemotherapy in addition to adjuvant endocrine therapy in patients younger than 50 with lymph-node negative breast cancer and OncotypeDx 21-gene Recurrence Scores (RS) between 16 and 25, no benefit was seen in women older than 50^4^. The RxPONDER trial randomized patients with 1–3 positive lymph nodes and RS 0–25 to either adjuvant endocrine therapy or endocrine therapy plus chemotherapy^5^. It also demonstrated improved IDFS with chemotherapy in premenopausal patients, or in patients 50 or younger, but no benefit was seen in older women^5^. In the MINDACT trial, a subset of HR+/HER2− patients with high clinical risk and low genomic risk (by the MammaPrint assay) were randomly assigned to receive adjuvant chemotherapy or not^6^. An exploratory analysis showed improved distant metastasis-free survival (DMFS) with chemotherapy compared to endocrine therapy alone in women younger than 50, but not in women older than 50^6^. In all three trials, the most frequently used endocrine therapy for premenopausal women was tamoxifen.

It is unclear what explains the interaction between age and adjuvant chemotherapy benefit. Age is difficult to separate from its association with menopausal status. The mean age of onset of menopause is 51 years in Western countries and by age 55 approximately 85% of women have undergone menopause^7,8^. Adjuvant chemotherapy in pre-menopausal women can induce menopause in an age-dependent manner^9,10^. The NSABP B-47 clinical trial showed that chemotherapy induced amenorrhea in pre-menopausal women is common but it is often discordant with hormone level measurements. In this study, 85% of patients were amenorrhoeic at 12 months after starting adjuvant chemotherapy but only 28 and 22% had postmenopausal estradiol levels at 12 and 24 months^11^. The SOFT and TEXT trials demonstrated that in premenopausal HR+ patients ovarian suppression plus an aromatase inhibitor is more effective than tamoxifen alone to improve recurrence-free survival^12,13^. Chemotherapy-induced menopause can therefore contribute to adjuvant chemotherapy benefit. However, younger patients also have more chemotherapy sensitive cancers. A pooled analysis of 9000 patients enrolled in neoadjuvant chemotherapy trials showed that the pathologic complete response (pCR) rate is significantly higher in the younger HR+/HER2− patients^14^.

In the past 20 years, three types of molecular features emerged that predict endocrine and chemotherapy sensitivities in early stage-breast HR+/HER2− cancer; (i) expression of estrogen receptor (ER) regulated genes is a measure of endocrine sensitivity and is associated with better prognosis^15^, (ii) proliferation, and (iii) immune infiltration related markers are independently associated with greater chemotherapy sensitivity in neoadjuvant chemotherapy trials^16–18^.

The goal of the current analysis was to compare differences in estrogen receptor (ER)-, proliferation-, and immune-related gene expressions, and somatic mutation patterns and mutation burden between younger (≤50 years of age) and older (≥55 years) patients with HR+/HER2− breast cancer that could explain the chemotherapy benefit in younger women. These age cohorts were selected because the ≤50 group is highly enriched in pre-menopausal women and represents the group where all the chemotherapy benefit accrues, whereas the ≥55 group is almost entirely composed of post-menopausal women^8^. We further restricted our analysis to the subset of patients who were in the lower 80% range of in silico RS distribution to mimic the RxPONDER and TAILORX populations that excluded women with RS > 25.

## Results

### Patient characteristics

Patient and tumor characteristics, including molecular subtype distribution, and available treatment information are presented in Table 1. The median ages of the younger and older patients ranged between 45–46 and 66–69 years across the datasets.

**Table 1:** Demographics and characteristics of ER+/HER2− cases in five cohorts.

Characteristic	TCGA cohort			Microarray-cohort-A			Microarray-cohort-B			METABRIC			SCAN-B		
	Younger	Older	*P* value*	Younger	Older	*P* value*	Younger	Older	*P* value*	Younger	Older	*P* value*	Younger	Older	*P* value*
	(age ≤ 50, *n* = 202)	(age ≥ 55, *n* = 461)		(age ≤ 50, *n* = 281)	(age ≥ 55, *n* = 584)		(age ≤ 50, *n* = 162)	(age ≥ 55, *n* = 447)		(age ≤ 50, *n* = 157)	(age ≥ 55, *n* = 710)		(age ≤ 50, *n* = 305)	(age ≥ 55, *n* = 1331)	
Median age (range)	45 (26–50)	66 (55–90)		46 (24–50)	66 (55–94)		45 (24–50)	66 (55–88)		45 (26–50)	69 (55–92)		46 (24–50)	68 (55–95)	
Menopausal Status (%)			<0.001									<0.001			
Pre	130 (71.3)	6 (1.5)		na.	na.		na.	na.		157 (100.0)	0 (0.0)		na.	na.	
Indeterminate	6 (3.5)	8 (2.0)		na.	na.		na.	na.		0 (0.0)	0 (0.0)		na.	na.	
Post	20 (11.9)	367 (91.1)		na.	na.		na.	na.		0 (0.0)	710 (100.0)		na.	na.	
Unknown	30 (13.3)	22 (5.4)		na.	na.		na.	na.		0 (0.0)	0 (0.0)		na.	na.	
Ethnicity, *n* (%)			0.02			0.25			<0.05						
White	122 (76.7)	316 (85.2)		106 (37.7)	254 (43.5)		44 (27.2)	112 (25.1)		na.	na.		na.	na.	
African American	22 (13.8)	36 (9.7)		3 (1.1)	2 (0.3)		7 (4.3)	10 (2.2)		na.	na.		na.	na.	
Asian	8 (5.0)	10 (6.9)		1 (0.4)	1 (0.2)		3 (1.8)	1 (0.2)		na.	na.		na.	na.	
Unknown	7 (4.4)	9 (2.4)		171 (60.8)	327 (56.0)		108 (66.7)	324 (72.5)		na.	na.		na.	na.	
Tumor stage, *n* (%)			0.02			0.83			0.99			0.07			0.98
T1 & 2	108 (67.9)	283 (76.3)		60 (21.4)	91 (15.6)		23 (14.2)	33 (7.4)		121 (77.1)	481 (67.7)		3 (1.0)	16 (1.2)	
≥T3	50 (31.4)	88 (23.7)		112 (39.8)	159 (27.2)		67 (41.4)	96 (21.5)		5 (3.2)	35 (5.0)		301 (99.0)	1313 (98.8)	
Unknown	1 (0.6)	0 (0.0)		109 (38.8)	334 (57.2)		72 (44.4)	318 (71.1)		31 (19.7)	194 (27.3)				
Lymph node, *n* (%)			<0.001			0.04			0.37			<0.001			0.06
Positive (≥1 positive)	94 (59.1)	136 (36.6)		54 (19.2)	147 (25.2)		68 (42.0)	180 (40.3)		45 (71.3)	328 (53.8)		120 (39.3)	429 (32.3)	
Negative (0 positive)	52 (32.7)	172 (46.4)		227 (80.8)	423 (72.4)		46 (28.4)	152 (34.0)		112 (28.7)	382 (46.2)		177 (58.0)	863 (64.8)	
Unknown	13 (8.2)	63 (17.0)		0 (0.0)	14 (2.4)		48 (29.6)	115 (25.7)		0 (0.0)	0 (0.0)		8 (2.6)	39 (2.9)	
Histological grade, *n* (%)						0.06			0.05			0.18			0.06
Grade 1 or 2	na.	na.		200 (71.2)	426 (72.9)		99 (35.2)	243 (41.6)		110 (70.1)	459 (64.6)		250 (82.0)	1137 (85.4)	
Grade 3	na.	na.		66 (23.5)	107 (18.3)		47 (16.7)	111 (19.0)		43 (27.4)	210 (29.6)		50 (16.4)	187 (14.1)	
Unknown	na.	na.		15 (5.3)	51 (8.7)		135 (48.1)	230 (39.4)		4 (2.5)	41 (5.8)		5 (1.6)	7 (0.5)	
Adjuvant treatment, *n* (%)						<0.001			<0.001			<0.001			<0.001
No adjuvant treatment	na.	na.		168 (59.8)	267 (45.7)		17 (6.0)	30 (5.1)		76 (48.4)	164 (23.0)		26 (11.1)	148 (8.5)	
Endocrine treatment only	na.	na.		18 (6.4)	238 (40.8)		13 (4.6)	184 (31.5)		53 (33.8)	517 (72.5)		114 (74.1)	986 (37.4)	
Chemotherapy + Endocrine	na.	na.		62 (22.1)	46 (7.9)		55 (19.6)	35 (6.0)		27 (17.2)	29 (4.1)		163 (14.3)	190 (53.4)	
Unknown	na.	na.		33 (11.7)	33 (5.6)		196 (69.8)	335 (57.4)		1 (0.6)	0 (0.0)		2 (0.5)	7 (0.6)	
Subtype, *n* (%)			0.11			0.018			0.05			<0.001			0.007
Basal	0 (0.0)	2 (0.5)		1 (0.4)	0 (0.0)		0 (0.0)	1 (0.2)		1 (0.6)	3 (0.4)		4 (1.3)	13 (1.0)	
Her2	0 (0.0)	0 (0.0)		13 (4.6)	16 (2.7)		11 (6.8)	20 (4.5)		4 (2.5)	9 (1.3)		5 (1.6)	12 (0.9)	
LumA	127 (79.9)	299 (80.6)		113 (40.2)	228 (39.0)		52 (32.1)	154 (34.5)		103 (65.6)	417 (58.7)		246 (80.7)	974 (73.2)	
LumB	19 (11.9)	55 (14.8)		82 (29.2)	225 (38.5)		63 (38.9)	212 (47.4)		22 (14.0)	221 (31.1)		28 (9.2)	233 (17.5)	
Normal	13 (8.2)	15 (4.0)		72 (25.6)	115 (19.7)		36 (22.2)	60 (13.4)		27 (17.2)	56 (7.9)		22 (7.2)	99 (7.4)	

* Chi-squared test *p* value; na means not available.

### Differences in ER signaling, cell proliferation, and immune infiltration

**Fig. 1:**
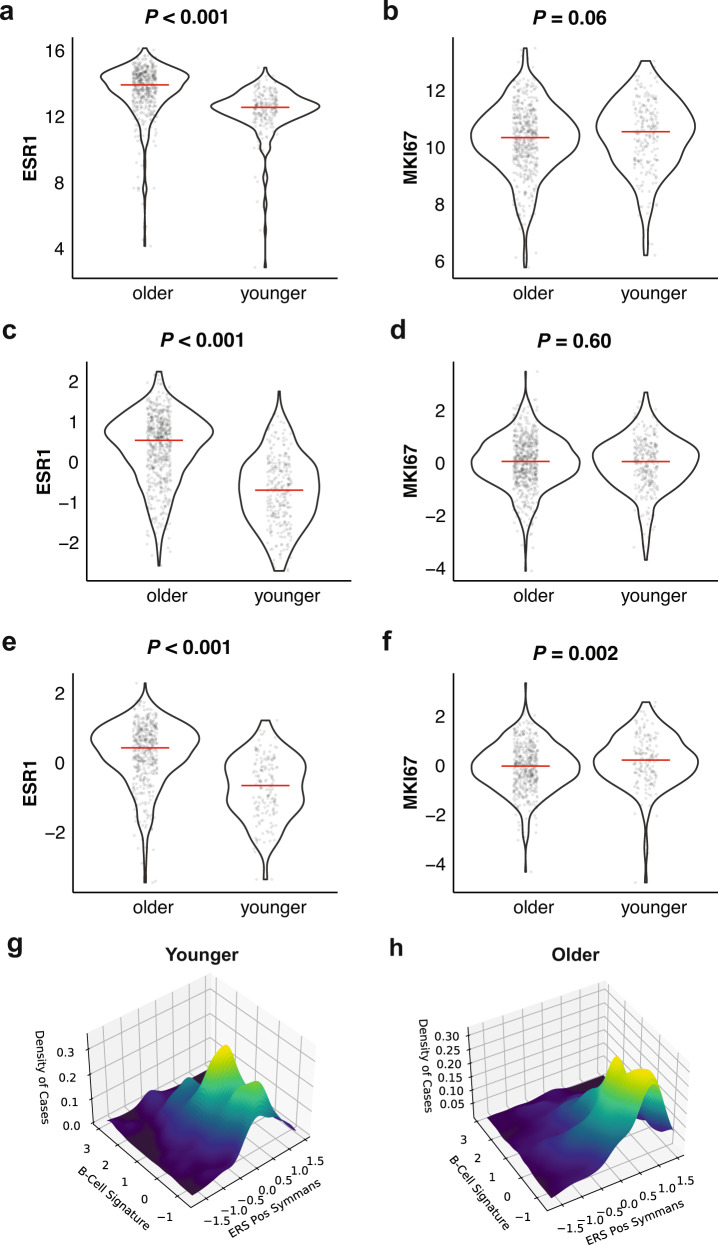
Expression of estrogen receptor (*ESR1*) and proliferation marker (*MKI67*) in older and younger ER+/HER2− breast cancer patients. **a** *ESR1* mRNA and **b** *MKI67* mRNA expression in TCGA cohort; **c** *ESR1* and **d** *MKI67* expression in Microarray Cohort-A; **e** *ESR1* and **f** *MKI67* expression in Microarray Cohort-B. P-values were estimated by the two-side Wilcoxon rank-sum test. Each dot represents a patient, the solid horizontal line indicates the median expression in each age group. Relationship between ERS-pos and B-Cell gene signatures in women 50 years of age or younger (**g**) and 55 years of age or older (**h**). The values from negative to positive in the *X* and *Y* axes denote increasing signature expression. *Z*-axis indicates case density (i.e., number of patients), color coding reflects increasing case density from dark blue to yellow.

*ESR1* mRNA expression was significantly lower in younger women in all cohorts (*P* < 0.001; Fig. 1a, c, e, Supplementary Fig. 1). Lower mRNA expression in bulk RNA analysis could be due to either fewer ER-positive cancer cells, that could be reflected by lower ER percent positivity by immunohistochemistry (IHC), or to lower ER mRNA expression within ER-positive cells. To distinguish between these two possibilities, we plotted age distribution in ten IHC percent positivity brackets from 1 to 10% to >90% in increments of 10 in the TCGA data where this information was available (*n* = 338). We observed no statistically significant correlation between age and increasing ER IHC percent positivity (*τ* = 0.036, *P* < 0.19, Supplementary Fig. 2a). Overall, *ESR1* mRNA expression increased as IHC percent positivity increased (*τ* = 0.27, *P* < 0.0001), reaching a plateau after > 40% (Supplementary Fig. 2b). ESR1 mRNA expression showed positive association with age at diagnosis (Spearman coefficient = 0.41, *P* < 0.0001) (Supplementary Fig. 2c). A regression model of *ESR1* mRNA expression using age and IHC positivity showed contribution of both parameters but a larger effect size of age (standardized beta 0.365) than percentage of IHC positivity (standardized beta 0.215). This suggests that the overall lower *ESR1* mRNA expression in younger patients is primarily driven by lower ESR1 mRNA levels in ER positive cancer cells.

Next, we assessed the expression of four gene signatures that are positively associated with endocrine therapy sensitivity including a 4-gene ERS^19^, a 7-gene ERS-Lum^19^, a 106-gene ERS-Pos signature^15^, and a 59-gene ERS-Neg signature^15^ which is negatively associated with ER expression and endocrine sensitivity^15^. Both in the TCGA and in the Metabric cohort, the ERS, ERS-Lum, and ERS-Pos signatures were all significantly lower (FDR < 0.03) while the ERS-Neg signature was higher (FDR < 0.001) in younger patients (Table 2). Similarly, in both microarray cohorts, and in the SCAN-B-cohort, the ERS-Pos signature was lower and the ERS Neg signature was higher in the younger age group (FDR < 0.002; Table 2). The two smaller signatures, ERS and ERS-Lum, showed nominally lower expressions in younger patients in cohort-A without reaching statistical significance. In cohort-B, ERS showed lower expression in young patients whereas ERS-Lum was similar between age groups (Table 2). Overall, these results indicate not only downregulation of ESR1 mRNA expression but also lower ER-associated gene expression in ER positive cancers of younger compared to older patients.

**Table 2:** Estrogen receptor and immune and proliferation-related signatures in younger and older breast cancer patients.

		Signature	log2 fold change	Mean in younger	Mean in older	*P* value	FDR
TCGA	Estrogen receptor	ERS Neg Symmans	0.52	0.26	−0.26	<0.001	<0.001
		ERS Pos Symmans	−0.21	0.09	0.30	<0.001	<0.001
		ERS luminal	−0.17	0.14	0.31	<0.001	<0.001
		ERS	−0.14	0.20	0.34	0.004	0.005
	Immune	B cell	0.34	0.25	−0.09	<0.001	<0.001
		T cell	0.35	0.19	−0.16	<0.001	<0.001
		Mast cell	0.25	0.29	0.04	0.003	0.004
		TIS	0.26	0.09	−0.18	0.002	0.003
		Dendritic cell	0.15	−0.04	−0.19	0.13	0.22
	Proliferation	MKS	0.07	−0.31	−0.37	0.46	0.46
Cohort-A	Estrogen receptor	ERS Neg Symmans	0.33	0.22	−0.11	<0.001	<0.001
		ERS Pos Symmans	−0.31	−0.21	0.10	<0.001	<0.001
		ERS luminal	−0.13	−0.09	0.04	0.08	0.14
		ERS	−0.07	−0.05	0.02	0.21	0.31
	Immune	B cell	0.20	0.13	−0.06	0.001	0.004
		Mast cell	0.16	0.11	−0.05	0.002	0.004
		T cell	0.13	0.09	−0.04	0.08	0.14
		TIS	0.04	0.02	−0.01	0.61	0.66
		Dendritic cell	−0.03	−0.02	0.01	0.59	0.66
	Proliferation	MKS	0.03	0.02	−0.01	0.71	0.71
Cohort-B	Estrogen receptor	ERS Neg Symmans	0.27	0.20	−0.07	<0.001	0.002
		ERS Pos Symmans	−0.22	−0.16	0.06	0.002	0.005
		ERS Luminal	0.01	0.01	0.00	0.95	0.95
		ERS	−0.10	−0.07	0.03	0.18	0.25
	Immune	B cell	0.51	0.38	−0.14	<0.001	<0.001
		T cell	0.41	0.30	−0.11	<0.001	<0.001
		TIS	0.20	0.15	−0.05	0.02	0.03
		Dendritic cell	0.14	0.10	−0.04	0.02	0.04
		Mast cell	0.01	0.01	0.00	0.73	0.88
	Proliferation	MKS	−0.01	0.00	0.00	0.88	0.95
METABRIC	Estrogen receptor	ERS Neg Symmans	0.63	0.51	−0.11	<0.001	<0.001
		ERS Pos Symmans	−0.29	−0.24	0.05	<0.001	0.001
		ERS luminal	−0.21	−0.18	0.04	0.02	0.03
		ERS	−0.44	−0.36	0.08	<0.001	<0.001
	Immune	B cell	0.22	0.18	−0.04	<0.001	<0.001
		T cell	0.20	0.17	−0.04	0.004	0.009
		TIS	0.09	0.08	−0.02	0.28	0.28
		Dendritic cell	0.06	0.05	−0.01	0.19	0.22
		Mast cell	0.21	0.18	−0.04	0.01	0.01
	Proliferation	MKS	−0.11	−0.09	0.02	0.20	0.22
SCAN-B	Estrogen receptor	ERS Neg Symmans	0.33	0.27	−0.06	<0.001	<0.001
		ERS Pos Symmans	−0.09	−0.07	0.02	0.06	0.10
		ERS luminal	−0.01	−0.01	0.00	0.69	0.69
		ERS	−0.09	−0.08	0.02	0.06	0.10
	Immune	B cell	0.31	0.25	−0.06	<0.001	<0.001
		T cell	0.25	0.20	−0.05	<0.001	<0.001
		TIS	0.13	0.11	−0.02	0.16	0.25
		Dendritic cell	−0.22	−0.18	0.04	<0.001	0.002
		Mast cell	0.09	0.07	−0.02	0.25	0.33
	Proliferation	MKS	0.08	0.07	−0.01	0.32	0.38

mRNA expression of the *MKI67* gene, that codes for the Ki67 proliferation marker, was similar between age groups in TCGA and microarray cohort-A, but was slightly but statistically significantly higher in the younger patients in microarray cohort-B (Fig. 1b, d, f and Supplementary Fig. 1). The expression of a 12-gene mitotic kinase gene signature (MKS), that has been associated with worse prognosis in HR positive breast cancers and higher sensitivity to neoadjuvant chemotherapy^14^, did not differ statistically significantly between the age groups in all cohorts (Table 2). However, the most highly proliferative tumors with the highest 20% of in silico RS were not included in this analysis by design.

Next, we assessed 4 different immune cell signatures^20^ and a tumor inflammation signature^21^ that were previously shown to predict response to chemotherapy and immune checkpoint inhibitor therapy (Table 2). In the TCGA, B-cell, T-cell, Mast-cell, and TIS signatures were significantly higher, the dendritic signature only showed nominally increased expression (FDR = 0.22). In the microarray Cohort-A, B cells and mast cells were significantly higher, the T cell and TIS signatures showed a trend for higher expression. In Cohort-B, T cells, B cells, TIS, and dendritic cells signatures were significantly higher in younger patients (Table 2). We also evaluated these gene signatures in the METABRIC and SCAN-B data sets and found similar associations (Table 2). We also performed an immune cell composition analysis in the TCGA data using the ConsensusTME method^22^. Consistent with the gene signature results, younger patients had higher levels B cells, Cytotoxic cells, Endothelial, Fibroblasts, Plasma cells, CD4 T cell, CD8 T cells, and T regulatory cell markers (Supplementary Fig. 3).

Next, we assessed correlation between the *ESR1*, *MKI67* expression, and the 10 gene signatures in Table 2. The *MKI67* expression and MKS signature, and ESR1 expression and the ERS-Pos gene signature were each highly correlated. The correlation between *ESR1* and the other ER-related gene signatures was less strong. Among the immune signatures, the T cell, B cell, and TIS signatures showed the highest co-expression. The ER-related and immune signatures showed moderate negative correlation in all 3 data sets (Pearson correlation coefficients −0.24, −0.31, −0.25) suggesting independent predictive functions (Supplementary Fig. 4). The distributions of the B cell and ERS-Pos signatures in the TCGA cohort are shown on Fig. 1g, h and illustrate that in the age ≤50 group, three patient populations are intermixed including those with immune-intermediate/ER-intermediate (largest subset), immune-low/ER-intermediate, and immune-high/ER-low (smallest subset) cancers, while in the older age group the immune-low/ER-high cancers are predominant.

### Differentially expressed genes and pathways between age groups

**Fig. 2:**
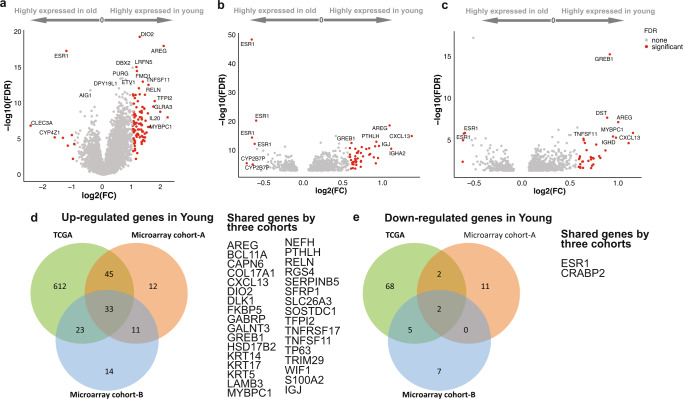
Differentially expressed genes between younger versus older patients. Volcano plots indicate the log2 fold change (FC) and FDR of differentially expression analysis in three cohorts, including **a** TCGA cohort; **b** microarray cohort-A; **c** microarray cohort-B. *P*-values were generated by Student’s t-test and corrected by Benjamini–Hochberg FDR. Red dots indicated genes meet criteria of fold change ≥ 1.50 or ≤ 0.67 and FDR < 0.05 (significant), and other genes were marked as gray (none, not significant). Top enriched genes with fold change ≥ 1.50 or ≤ 0.67 and FDR < 1e−05 were labeled with official gene symbols. **d**, **e** Venn diagram shows the number of upregulated (**d**) and downregulated (**e**) genes identified in the TCGA that also showed up- or downregulation in the other two data sets.

In the TCGA, we identified 713 up- and 77 downregulated genes in younger patients (Fig. 2a and Supplementary Table 1). In microarray cohorts A and B, we found 122 and 95 upregulated and 15 and 14 downregulated genes, respectively (Fig. 2b, c, Supplementary Tables 2 & 3, and Supplementary Fig. 5). Thirty-one upregulated genes in younger patients were shared in all three analyses (Fig. 2d, e). Twenty-five and 11 of the 31 overlapped DEGs were also upregulated in young patients in SCAN-B and METABRIC cohort, respectively (Supplementary Table 4). *ESR1* and *CRABP2* were down-regulated in both SCAN-B and METABRIC cohorts (Supplementary Table 4). In gene set enrichment analysis, 22 biological pathways showed differential expression by age in TCGA; 7 were immune and inflammation related, the others represented estrogen, K-ras, and hedgehog signaling, epithelial mesenchymal transition, angiogenesis, and apical junction/apical surface pathways (Supplementary Table 5).

### Comparison of somatic mutations and copy number variations (CNV) in younger versus older patients in TCGA

**Fig. 3:**
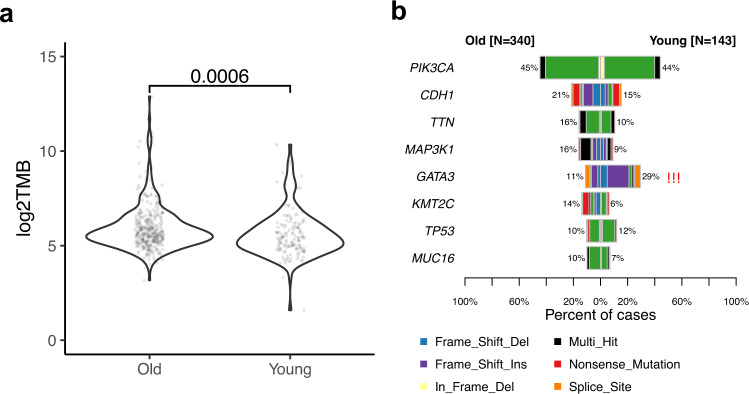
Somatic mutation profiles between younger versus older TCGA patients. **a** Tumor mutation burden. *P*-values from Wilcoxon rank-sum test. **b** Somatic mutation frequency. Only genes with mutation frequency > 5% in both groups are shown. ***Fisher’s exact test *p* value < 0.0001.

The somatic mutation burden was significantly higher in older patients (*P* < 0.0001; Fig. 3a), consistent with age-related accumulation of mutations^23^. At gene level, 13 genes had mutation frequencies ≥ 5% and only *GATA3* showed a significantly higher mutation frequency in younger patients (26% versus 12%, *P* < 0.0001; Fig. 3b). In multivariate logistic regression analysis, luminal B tumors were associated with the enrichment of *GATA3* mutations (*P* = 0.011, odds ratio = 2.18), younger patients also had higher rate of *GATA3* mutations (*P* < 0.0001, odds ratio = 3.15). These results are consistent with an earlier report that showed *GATA3* mutation enrichment in luminal B cancers from young women^24^.

We also compared the CNV gain and loss of 705 Catalog Of Somatic Mutations In Cancer (COSMIC) genes^25^. We identified high rate of CNV gain of *ESR1, LATS1, ARID1B, SGK1*, and *MYB* genes (odds ratio > 8.5, FDR < 0.05) in old patients (Supplementary Table 6). Young patients have a higher rate of CNV loss of *ESR1* gene (odds ratio = 0.45, FDR = 0.03, Supplementary Table 6). In addition to *ESR1*, we identified 19 and 29 genes have higher rate of CNV loss in young and old patients, respectively (Supplementary Table 6).

## Discussion

In independent data sets including *n* = 4507 ER+/HER2− breast cancers, we found that cancers in patients 50 or younger have lower expression of *ESR1* and ER-related genes and higher expression of immune related genes. Increasing ER expression with older age has been described in earlier studies that analyzed all breast cancer subtypes together^26^. A significant linear relationship between increasing age and *ESR1* mRNA expression was also seen in luminal-A and -B breast cancers^27^. The biological reasons behind this phenomenon are unclear. In normal breast epithelium in premenopausal women, the ER expression fluctuates during the menstrual cycle, and ER expression is highest during the follicular phase^28,29^. Based on this observation, one would expect higher average ER expression in premenopausal women, however, we found the opposite. We hypothesize that ER expression in breast epithelial cells, and in cancers that arise from them, may increase as estrogen levels decrease with aging due to a feedback loop. Indeed, several studies showed increased ER expression in normal breast epithelium with increasing age^30,31^.

The clinical relevance of lower *ESR1* and ER related gene expression in cancers of younger women is uncertain. However, ER-associated genes are components of all clinically validated multi-gene prognostic signatures^32^, and higher expression levels are associated with better prognosis with adjuvant endocrine therapy^33^. Higher ER-associated gene expression is also associated with longer PFS and OS in metastatic breast cancer treated with endocrine therapy^34^. These results suggest that lower *ESR1* and ER-related gene expression in younger women may indicate lower endocrine sensitivity. Intensifying endocrine therapy could maximize benefit, which is consistent with clinical trial results that demonstrated ovarian suppression plus tamoxifen, or exemestane, is more effective than tamoxifen alone to improve recurrence-free survival in premenopausal women.

The higher immune gene expression in younger HR+/HER2− breast cancer patients compared to older patients has not previously been reported. The cause of the higher immune infiltration is unknown. Somatic mutation burden that could increase neoantigen load was lower in younger patients. The gene expression data suggests an important role for *CXCL13* that was the most highly and consistently overexpressed chemokine in cancers from younger women. *CXCL13* is secreted by dendritic and endothelial cells, and is a powerful B cell attractant, that can also activate helper T cells^35^. High expression of *CXCL13* is predictive of better survival in HR+/HER2− breast cancer patients treated with adjuvant chemotherapy^36^, and is associated with higher pathologic complete response rate after neoadjuvant chemotherapy in HR+ breast cancers^17^. These observations suggest that HR+/HER2− breast cancer in younger patients may have higher chemotherapy sensitivity due to greater immune infiltration in the tumor microenvironment than cancers in older women, even if proliferation related predictive markers are similar. When we examined immune and ER related gene expression distributions jointly, we found 3 distinct sub-populations among younger women; (i) immune-high/ER-low, (ii) immune-intermediate/ER-intermediate, and (iii) immune-low/ER-intermediate cancers. The impact of adjuvant chemotherapy is likely different in these different subgroups. We hypothesize that in immune-high/intermediate and ER-low/intermediate cancers the cytotoxic effect drives the benefit, whereas in immune-low/ER-intermediate cancers chemotherapy-induced ovarian suppression plays a more important role. These observations add to the already existing literature that described general molecular differences between breast cancers in younger and older women including elevated integrin/laminin and EGFR and TGFβ signaling and numerous age-associated genes^37–39^. To increase our ability to identify differences between pre- and post-menopausal ER+ breast cancers our analysis focused on cancers from woman < 50 and >55 years of age groups and excluded the perimenopausal age group 50 to 55. We further restricted our analysis by excluding cases with the highest 20% of in silico RS. This is an important feature of our analysis that has impacted the findings, unlike all previous studies that find higher prevalence of luminal B cancers in younger women, our comparison cohorts were balanced for luminal A and B subtypes. This indicates that the higher chemotherapy benefit is not due to higher proportion of Luminal B cancers among premenopausal women with Recurrence Score <26. Finally, our purpose was to examine differences, if they exist, in carefully selected clinically validated biologic features that predict for chemotherapy and endocrine therapy sensitivity so that we could generate a hypothesis of why younger patients benefit more from chemotherapy.

This study has limitations. We were unable to assess the interaction between adjuvant treatments, molecular features and survival in the young women due to lack of patient specific treatment information in our datasets and lack of randomization. However, we describe a testable hypothesis that could be examined in future clinical trials prospectively, or retrospectively, when gene expression data becomes available from samples of the TAILORx or RxPONDER trials. We describe biological features that are highly reproducible across independent datasets and across different mRNA quantification platforms which implies that these robust gene expression features could be captured by standardized assays in the future.

Overall, our analysis suggests that both the cytotoxic and endocrine effects of adjuvant chemotherapy could contribute to the overall survival benefit seen in younger patients but the relative contributions of these effects may vary by the immune cell composition and ER expression of these cancers.

## Methods

### TCGA breast cancer cohort

mRNA expression, somatic mutation, and clinical data of 1085 primary breast cancer patients were obtained from TCGA (https://gdc.cancer.gov/about-data/publications/pancanatlas). The RNAseq expression matrix of Fragments per Kilobase of transcript per Million mapped reads (FPKM) was upper quantile normalized and subsequently log2 transformed. Percent ER positivity assessed by routine clinical immunohistochemistry (IHC) was available for 1037 cases^40^. We excluded the ER-negative (*n* = 238) and *HER2* amplified (*n* = 100) cases, and cases without ER information (*n* = 48). We assigned *HER2* status based on *HER2* mRNA expression that follows a bimodal expression pattern^41^. We used the Bayesian information criterion to find the number of components in the Gaussian mixture model and used GaMRed (http://cellab.polsl.pl/index.php/software?id=28)^42^ to select the optimal threshold value (normalized FPKM equal to 15.17) to define *HER2* gene overexpression. To mimick the TAILORx and RxPONDER populations we also excluded case with the top 20% in silico calculated RS score (*n* = 74). For final analysis, we grouped ER+/HER2− cancers (*n* = 530) into ≤ 50 (*n* = 159) or ≥ 55 years of age (*n* = 371) at diagnosis (Supplementary Fig. 6).

### Microarray cohorts

From publicly available Affymetrix microarray datasets we identified 2007 unique, previously untreated breast cancer samples that were (i) annotated with age, (ii) had raw MAS5 data deposited, and (iii) were ER+/HER2−^43^ (Supplementary Fig. 6). We assembled 27 Affymetrix U133A datasets from GEO (https://www.ncbi.nlm.nih.gov/geo/) and ArrayExpress (https://www.ebi.ac.uk/arrayexpress/) (E-TABM-158, GSE11121, GSE12276, GSE16391, GSE17907, GSE18864, GSE19615, GSE20194, GSE2034, GSE2109, GSE21653, GSE22035, GSE22513, GSE2603, GSE26971, GSE2990, GSE3494, GSE4611, GSE46184, GSE4922, GSE5327, GSE6532, GSE6532, GSE6596, GSE7390, GSE9195, MDA133) with no overlap to the RNA-Seq sample cohort from TCGA. We included only datasets with MAS5 data available (i.e., Individual sample level normalized expression data) without cohort-based normalization steps (e.g., RMA). A total of 3292 unique samples were annotated with age and had raw MAS5 data deposited. From these, we selected 2007 ER+/HER2− samples based on gene expression data as previously described^43^ (Supplementary Fig. 6). Supplementary Table 7 lists details for each sample including clinical information and a link to the corresponding expression data.

For the most accurate identification of differentially expressed genes, we aimed to assemble the most homogenous combined dataset with respect to technical bias and platform heterogeneity. To accomplish this, we used our previously described pipeline^44^ and designated this dataset as “Cohort A”. We calculated a technical comparability metric “C” which is the sum of squared normalized differences between dataset means and global means for all genes and considered datasets highly comparable if normalized C < 0.05. This resulted in 13 data sets including *n* = 1170 samples assigned into Cohort-A. For a second independent validation, we also combined all remaining datasets into Cohort B including *n* = 837 samples that correspond to data with grater technical heterogeneity (Supplementary Fig. 6).

From each cohort, we then excluded cases in the top 20% of highest in silico Recurrence score values to mimic a clinical cohort similar to that of TAILORx that included only patients with RS < 26. This resulted in *n* = 936 cases in Cohort A and *n* = 669 cases in Cohort B. For final analysis, we grouped ER+/HER2− cancers into ≤ 50 (*n* = 281 in cohort-A, *n* = 162 in cohort-B) versus ≥ 55 (*n* = 584 in cohort-A, *n* = 447 in cohort-B) years of age (Supplementary Fig. 6).

### METABRIC datasets

Normalized tumor mRNA expression data and the clinical metadata of 1908 breast cancer patients^45^ were download from www.cbioportal.org. We excluded 723 ER-negative or *HER2* amplified cases, 61 cases without ER or HER2 status, and 240 cases with the top 20% RS score. For final analysis, we grouped ER+/HER2− cancers (*n* = 867) into ≤ 50 (*n* = 157) or ≥ 55 years of age (*n* = 710) at diagnosis (Supplementary Fig. 6).

### SCAN-B datasets

Normalized tumor mRNA expression data and the clinical metadata of 2969 breast cancer patients were downloaded from the Gene Expression Omnibus (GEO) database (GSE96058)^46^ (Supplementary Fig. 6). ER status assessed by immunohistochemistry was available for 2,783 patients, and HER2 status reported by situ hybridization was available for 2868 patients. We excluded the ER-negative (*n* = 224) and *HER2* amplified (*n* = 378) cases, cases without ER (*n* = 199) or HER2 (101) status, and cases with top 20% RS score (*n* = 409). For final analysis, we grouped ER+/HER2− cancers (*n* = 1636) into ≤ 50 (*n* = 305) or ≥ 55 years of age (*n* = 1331) at diagnosis (Supplementary Fig. 6).

### Calculation of in silico recurrence score

We calculated an in silico recurrence score for each sample using the oncotypedx function of the *genefu* R library^47^. These scores approximate the clinical OncotypeDX RS but are not equivalent due to different dynamic ranges of the measurements. In clinical studies, 15–20% of cases submitted for OncotypeDx testing have RS > 25^48,49^. In the screening phase of TAILORx, 17% of patients had RS > 25. To approximate this distribution, we excluded patients with the top 20% of the highest continuous in silico recurrence scores.

### Molecular subtyping

Molecular subtype assignments of TCGA samples were obtained from Peng et al.^50^. To assign molecular subtypes to samples from the microarray cohorts we used the R package AIMS under R version 3.3.0^51^.

### Gene-expression signatures

To assess ER and Ki67 expression in the microarray data, we used the *ESR1* probe set 205225_at, and the average of four *MKI67* probe sets as previously described^43^. Ten mRNA expression signatures were obtained from literature including four estrogen-related signatures (e.g., ERS, ERS Luminal^19^, ERS Pos Symmans^15^, and ERS Neg Symmans^15^), four immune cell signatures (e.g., T Cell, B Cell, Mast Cell, Dendritic Cell^20^, and Tumor inflammation signature [TIS]^21^), and one proliferation signature (Mitosis Kinase Score, MKS^19^) (Supplementary Table 8). For each signature, we calculated the average normalized expression of the member genes and transformed to z-score across all cases in each cohort.

### Immune-cell composition analysis

Immune cell composition was estimated using the ConsensusTME^22^ method that estimates the contribution of 18 immune cell types to the tissue microenvironment. We used normalized TCGA mRNA expression data as input and select ssGSEA method for immune cell signature analysis with the ConsensusTME R package^22^.

### Differentially expressed genes

To identify differentially expressed genes (DEGs) in TCGA RNAseq data (representing 20,282 human genes), we calculated fold change and t-test *p*-value for each gene between younger and older cases. DEGs were defined as fold change ≥ 1.50 (i.e., upregulated) or ≤ 0.67 (i.e., downregulated) with Benjamini Hochberg corrected false discovery rate (FDR) < 0.05. To identify DEGs from Affymetrix microarray data, we applied the limma R package^52^. To avoid batch effects, we included the original Affymetrix source dataset as covariate. Identical fold change filters were used as for TCGA data.

### Gene set enrichment analysis

Log2 transformed fold changes of all 20,282 genes of TCGA samples were used as gene rank values to perform gene set enrichment analysis using the fgsea^53^ package in R using the hallmark gene set (*n* = 50) of the Molecular Signatures Database (MSigDB)^54^.

### Somatic mutation analysis

Somatic mutations which were available for 427 older and 183 younger TCGA breast cancer cases were obtained from the Multi-Center Mutation Calling in Multiple Cancers (MC3) dataset^55^. Somatic mutation burden was calculated as the total number of somatic mutations across all genes in each cancer. For comparison of gene level somatic mutation frequencies between age groups we only considered the nonsynonymous mutations, including missense, non-sense, frameshifting, in-frame shifting, or splice-site altering single-nucleotide changes or indels and statistical significance was assessed with Fisher’s exact test. A multivariate logistic regression model was used to evaluate the association of Luminal B subtype and age group with the mutation status of GATA3:

$${\it{GATA3}}\;{{{\mathrm{status}}}}\sim {{{\mathrm{Age}}}}\;{{{\mathrm{group}}}} + {{{\mathrm{Luminal}}}}\;{{{\mathrm{B}}}}\;{{{\mathrm{status}}}} + {{{\mathrm{Age}}}}\;{{{\mathrm{group}}}}\, \ast \,{{{\mathrm{Luminal}}}}\;{{{\mathrm{B}}}}\;{{{\mathrm{status}}}}$$

#### Association of ER status and age at diagnosis

We estimated the statistical significance of the trend of the ER IHC percentage categories with ESR1 mRNA expression and age at diagnosis using Jonckheere Terpstra (JT) trend analysis^56^. *P-*values were calculated using the “JonckheereTerpstraTest” function of “DescTools” R package^57^. Kendall’s tau (*τ*) coefficient was estimated to measure the increasing (positive value) or decreasing (negative value) trend for each trend analysis. We estimated the correlation between *ESR1* mRNA expression and age of diagnosis using Spearman’s rank correlation analysis.

### Copy number variation analysis

We obtained gene-level somatic CNV data of TCGA patients from the PanCanAtlas Aneuploidy study (https://gdc.cancer.gov/about-data/publications/pancanatlas)^58^. The CNVs of 25,128 genes of 513 ER+/HER2− patients were available. We focus on the 703 genes that overlapped with the COSMIC cancer gene list. The gene-level events indicate that the copy number gain/loss effect an entire chromosome arm or a specific genomic region that encodes gene. CNV was assessed with Affymetrix SNP 6.0 arrays^58^ and gene-level CNV values were generated by GISTIC^59^. A GISTIC call of +1 or +2 was considered a gain and −1 or −2 was considered a loss, and 0 as wild-type for association analysis in our study. The association of CNV gain or loss with the age group was assessed with Fisher’s exact test. Odds ratio larger than one were consider as CNVs enriched in old patients, and less than one means enriched in young patients.

### Statistical analysis

The Chi-squared test was used to compare categorical variables of patient characteristics. Wilcoxon rank-sum test was used to compare the expression signatures, and somatic mutation burden. *P*-values were adjusted for multiple comparisons using Benjamini–Hochberg method. A regression model of *ESR1* mRNA using age, ER IHC percentage categories, and their interaction was used to assess the contribution of both parameters. All analyses were performed in R version 3.6.1^51^.

### Reporting summary

Further information on research design is available in the Nature Research Reporting Summary linked to this article.

## Supplementary information

Supplementary Material

Reporting Summary

Supplemental Tables

## Data Availability

All the data that support the funding in this study are public available and Web links of those datasets are available in the Methods section, additional information can be provided by the authors upon reasonable request.

## Code availability

All codes for data cleaning and analysis are available at GitHub https://github.com/tao-qing/npjYoungVsOld.
