# Supplementary material for: Molecular differences between younger versus older ER-positive and HER2-negative breast cancers
Source: NPJ Breast Cancer. 2022 Nov 7;8:119. doi: 10.1038/s41523-022-00492-0 (PMC9640562; doi:10.1038/s41523-022-00492-0)
Supplement: Supplementary file 1 — Supplementary Material [file 41523_2022_492_MOESM1_ESM.pdf]

SUPPLEMENTARY MATERIALS

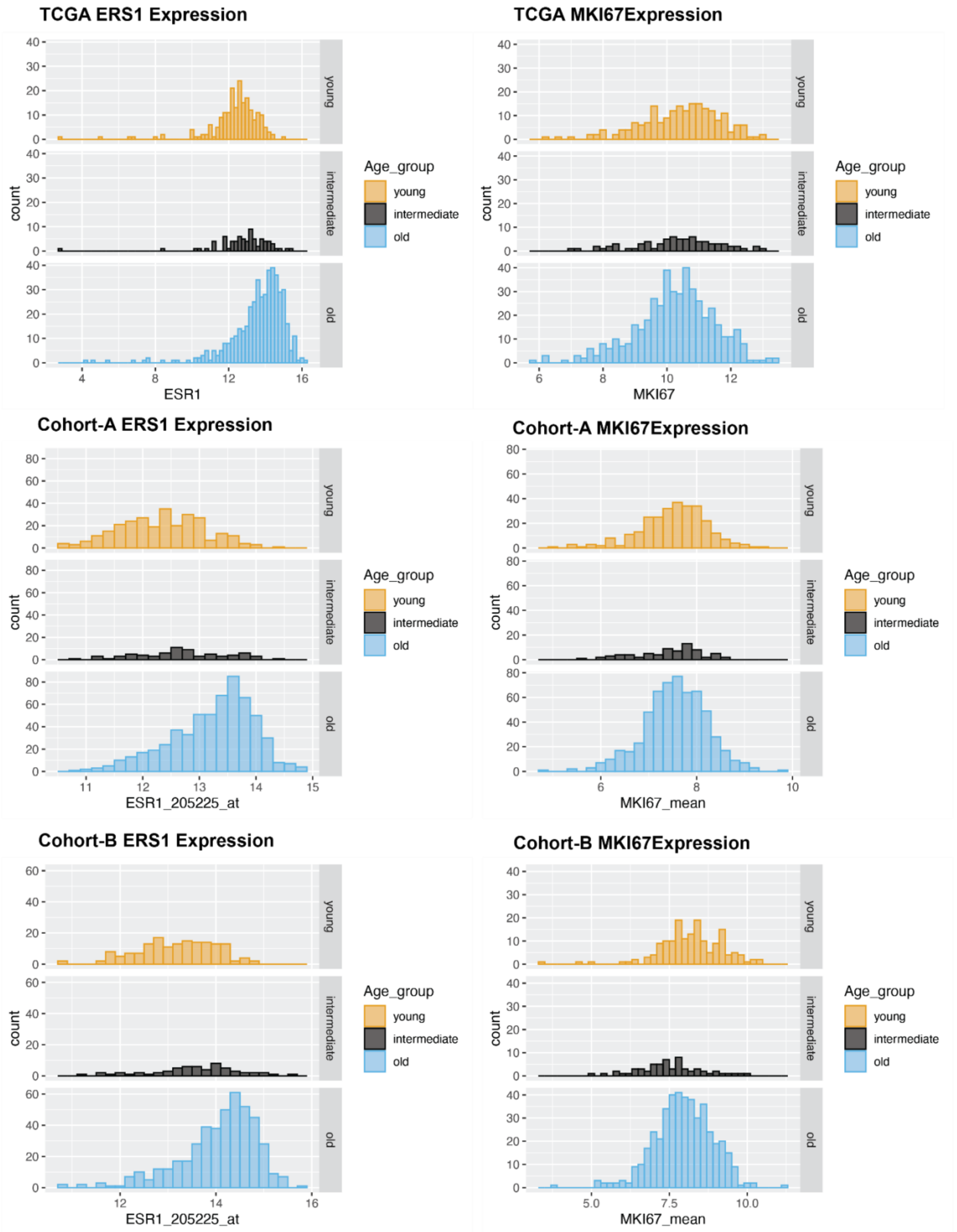

Supplementary Figure 1. Distribution of *ESR1* and *MKI67* mRNA expression in ER+/HER2- breast cancers in three cohorts. Yellow, dark, and blue indicate young (⩽50), intermediate (51-54), and old (⩾55) age groups, respectively. The X-axis shows gene-expression levels captured by a single probeset for *ESR1* and by the average expression of probes for *MKI67*.

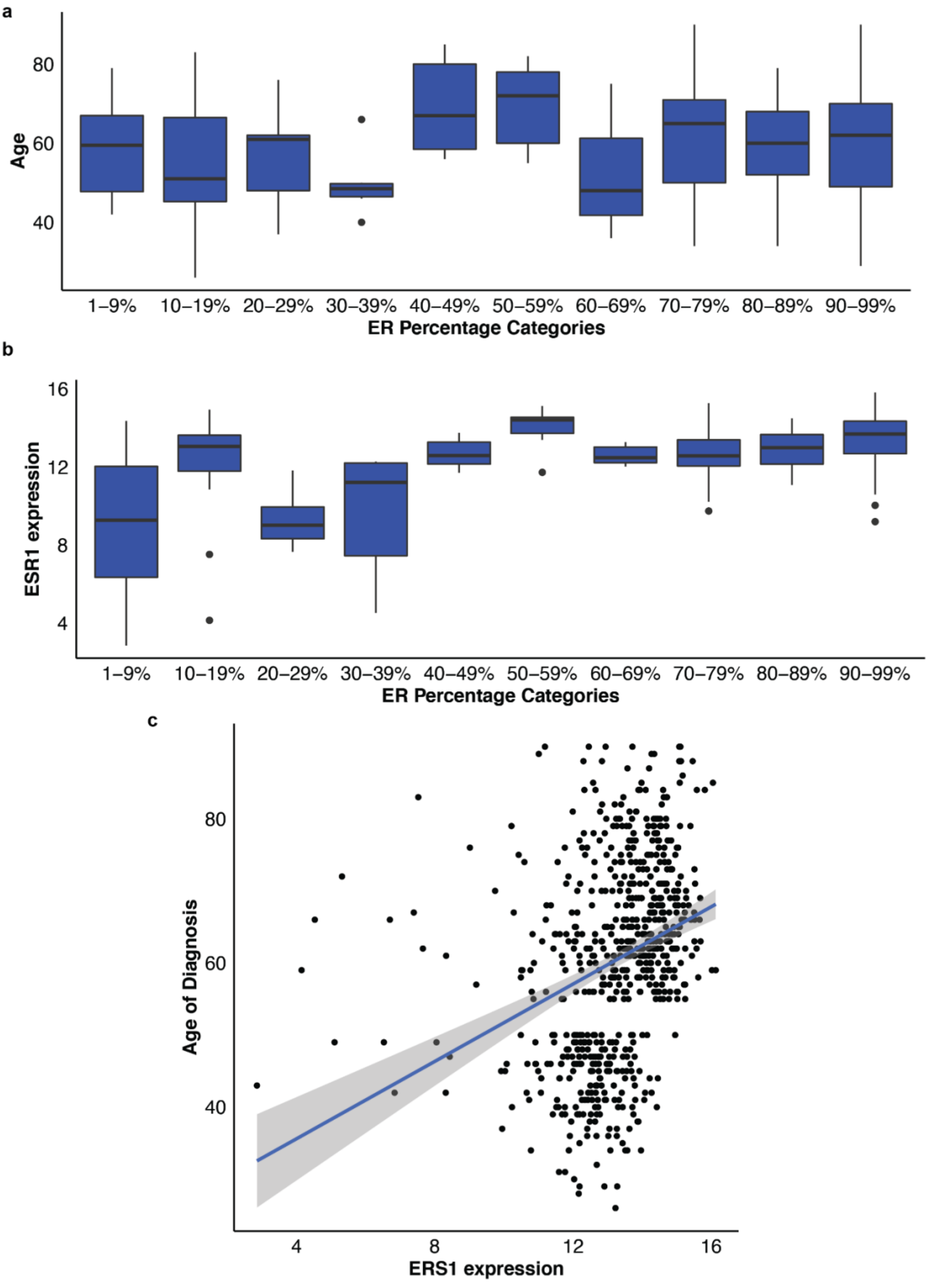

Supplementary Figure 2. *ESR1* mRNA expression and age distribution in different ER immunohistochemistry percent positive categories for 338 cases inTCGA ER+/HER2- for whom percent immunohistochemistry results were available. (a) Age versus ER immunohistochemistry percent positivity. (b) *ERS1* expression versus ER immunohistochemistry percent positivity. (c) Spearman correlation between *ERS1* mRNA expression versus age at diagnosis.

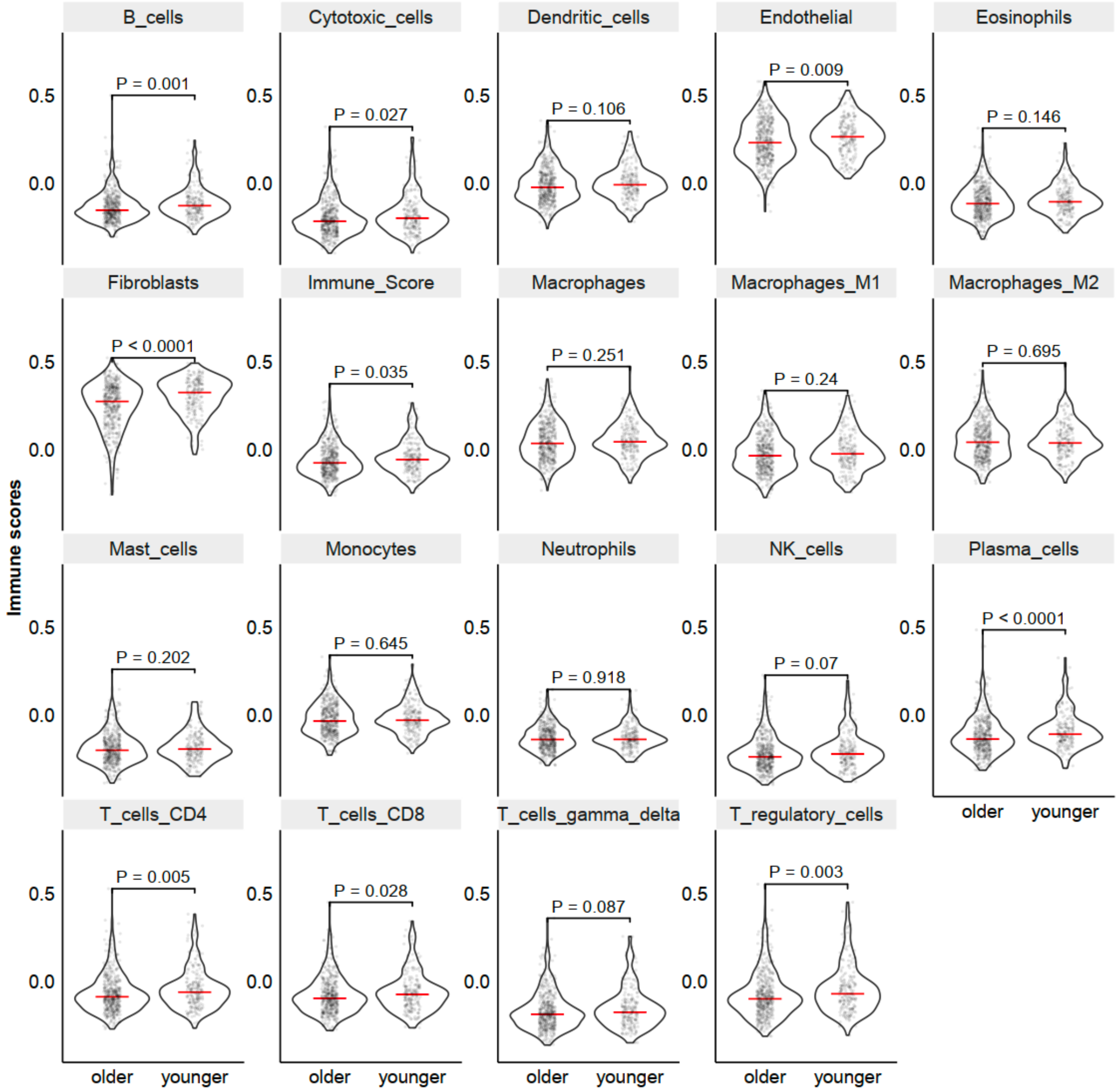

Supplementary Figure 3. Immune cell composition of TCGA patients calculated by ConsensusTME. P-values were estimated by the two-side Wilcoxon rank-sum test. Each dot represents a patient, the solid horizontal line indicates the median expression in each age group.

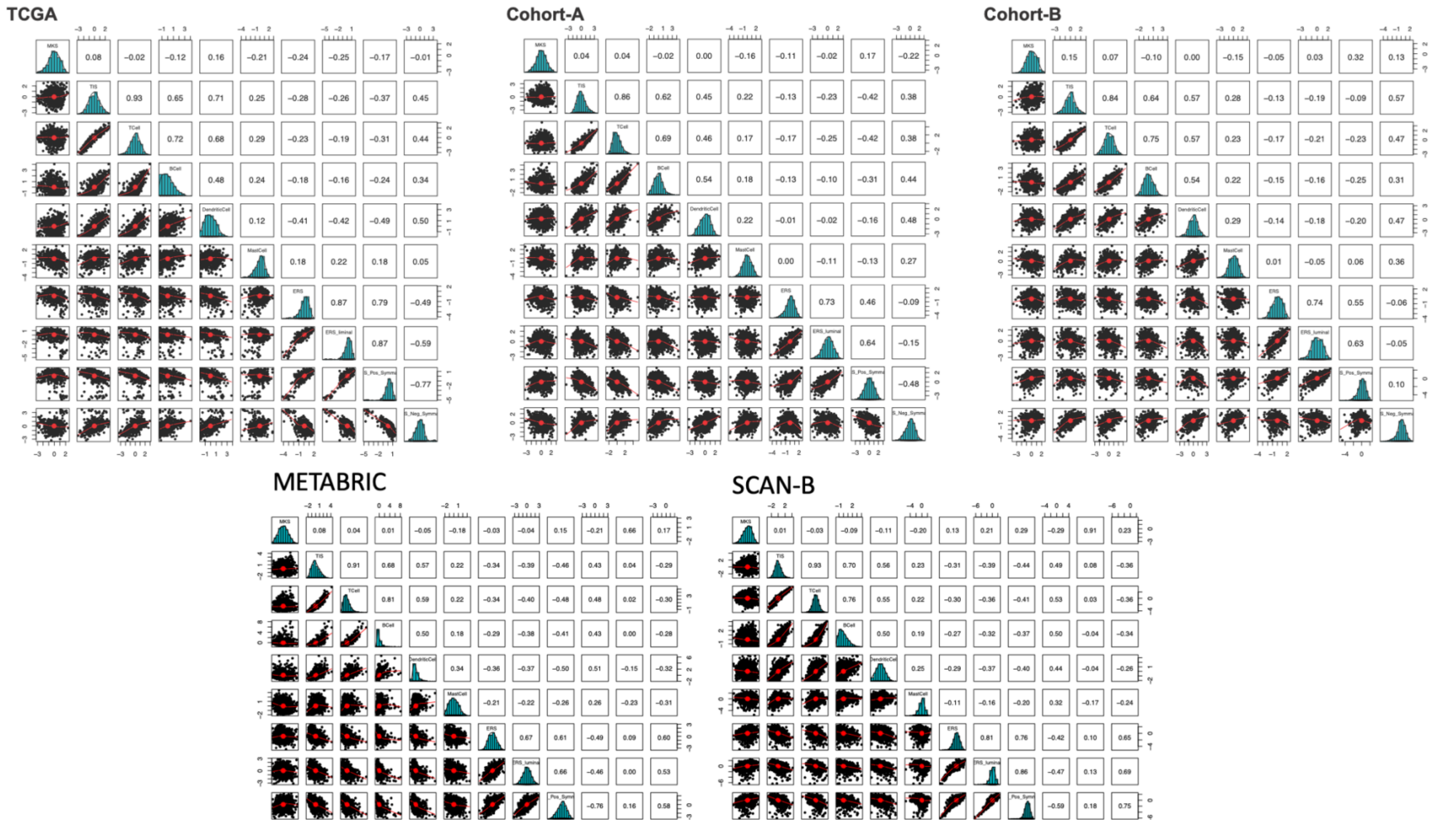

Supplementary Figure 4. Correlation between 10 gene signatures in the five distinct data cohorts. Cells below the diagonal show the scatter plots, red lines represent linear regression fits. Cells on the diagonal show the distribution histograms of each signature. Cells above the diagonal shown the Pearson correlation.

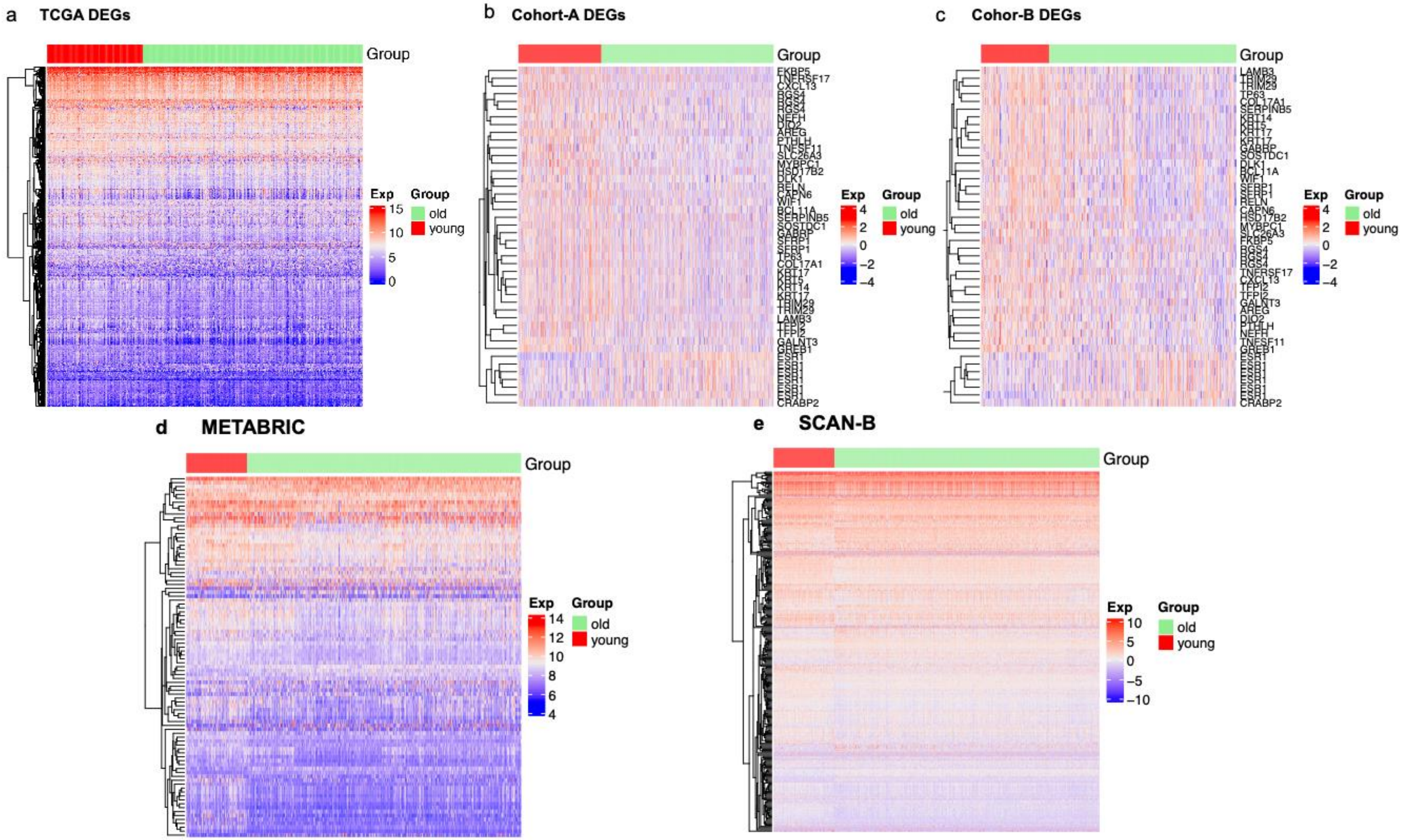

Supplementary Figure 5. Heatmaps show the expression profiles of differentially expressed genes in younger versus older cases in five cohorts. a. TCGA; **b.** Microarray Cohort-A; **c.** Microarray Cohort-B; **d.** METABRIC; **e.** SCAN-B. X- and Y-axes represent differently expressed genes and samples, respectively. The top color bars indicate young (red) and old (yellow) age groups. Heatmap color from blue to red indicates increasing gene-expression value. Differentially expressed genes were defined for each cohort as described in the methods section.

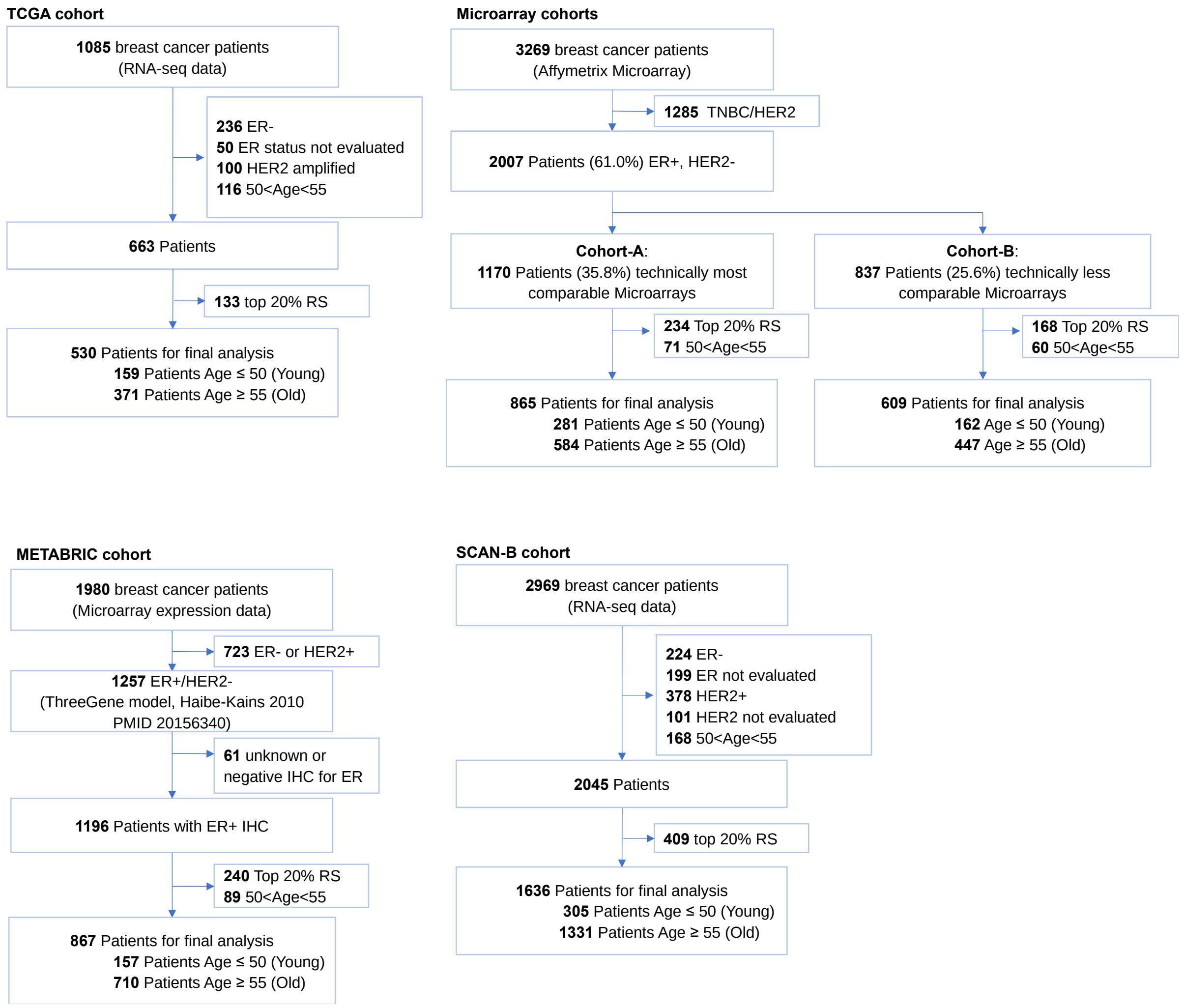

Supplementary Figure 6. Cancer cohorts in this study
