# Supplementary material for: Molecular differences between younger versus older ER-positive and HER2-negative breast cancers
Source: NPJ Breast Cancer. 2022 Nov 7;8:119. doi: 10.1038/s41523-022-00492-0 (PMC9640562; doi:10.1038/s41523-022-00492-0)
Supplement: Supplementary file 2 — Reporting Summary [file 41523_2022_492_MOESM2_ESM.pdf]

nature portfolio

Corresponding author(s): Lajos Pusztai

Last updated by author(s): Oct 6, 2022

Reporting Summary

Nature Portfolio wishes to improve the reproducibility of the work that we publish. This form provides structure for consistency and transparency in reporting. For further information on Nature Portfolio policies, see our Editorial Policies and the Editorial Policy Checklist.

Statistics

For all statistical analyses, confirm that the following items are present in the figure legend, table legend, main text, or Methods section.

n/a	Confirmed	
☐	☒	The exact sample size (*n*) for each experimental group/condition, given as a discrete number and unit of measurement
☐	☒	A statement on whether measurements were taken from distinct samples or whether the same sample was measured repeatedly
☐	☒	The statistical test(s) used AND whether they are one- or two-sided<br>*Only common tests should be described solely by name; describe more complex techniques in the Methods section.*
☐	☒	A description of all covariates tested
☐	☒	A description of any assumptions or corrections, such as tests of normality and adjustment for multiple comparisons
☐	☒	A full description of the statistical parameters including central tendency (e.g. means) or other basic estimates (e.g. regression coefficient) AND variation (e.g. standard deviation) or associated estimates of uncertainty (e.g. confidence intervals)
☐	☒	For null hypothesis testing, the test statistic (e.g. *F*, *t*, *r*) with confidence intervals, effect sizes, degrees of freedom and *P* value noted<br>*Give P values as exact values whenever suitable.*
☒	☐	For Bayesian analysis, information on the choice of priors and Markov chain Monte Carlo settings
☒	☐	For hierarchical and complex designs, identification of the appropriate level for tests and full reporting of outcomes
☐	☒	Estimates of effect sizes (e.g. Cohen's *d*, Pearson's *r*), indicating how they were calculated

Our web collection on statistics for biologists contains articles on many of the points above.

Software and code

Policy information about availability of computer code

Data collection	-mRNA expression, somatic mutation, and clinical data of 1,085 primary breast cancer patients were obtained from TCGA (https://gdc.cancer.gov/about-data/publications/pancanatlas).<br>-We assembled 27 Affymetrix U133A datasets from GEO (https://www.ncbi.nlm.nih.gov/geo/) and ArrayExpress (https://www.ebi.ac.uk/arrayexpress/) (E-TABM-158, GSE11121, GSE12276, GSE16391, GSE17907, GSE18864, GSE19615, GSE20194, GSE2034, GSE2109, GSE21653, GSE22035, GSE22513, GSE2603, GSE26971, GSE2990, GSE3494, GSE4611, GSE46184, GSE4922, GSE5327, GSE6532, GSE6532, GSE6596, GSE7390, GSE9195, MDA133) with no overlap to the RNA-Seq sample cohort from TCGA.<br>-Normalized tumor mRNA expression data and the clinical metadata of 1,908 METABRIC breast cancer patients were download from www.cbioportal.org.<br>-Normalized tumor mRNA expression data and the clinical metadata of 2,969 breast cancer patients were downloaded from the Gene Expression Omnibus (GEO) database (GSE96058)
Data analysis	All our analyses were performed using R-project (version 3.6.1).

For manuscripts utilizing custom algorithms or software that are central to the research but not yet described in published literature, software must be made available to editors and reviewers. We strongly encourage code deposition in a community repository (e.g. GitHub). See the Nature Portfolio guidelines for submitting code & software for further information.

Data

Policy information about availability of data

All manuscripts must include a data availability statement. This statement should provide the following information, where applicable:

- Accession codes, unique identifiers, or web links for publicly available datasets
- A description of any restrictions on data availability
- For clinical datasets or third party data, please ensure that the statement adheres to our policy

Data and code supporting the findings of this study are available in the Article and from the Supplementary Information files, additional information can be provided by the authors upon reasonable request.

Human research participants

Policy information about studies involving human research participants and Sex and Gender in Research.

Reporting on sex and gender	N/A
Population characteristics	Patients in our study were separated into two age groups: younger (<50 years of age) and older (>55 years).
Recruitment	We used public available dataset.
Ethics oversight	We used public available dataset.

Note that full information on the approval of the study protocol must also be provided in the manuscript.

Field-specific reporting

Please select the one below that is the best fit for your research. If you are not sure, read the appropriate sections before making your selection.

☒ Life sciences ☐ Behavioural & social sciences ☐ Ecological, evolutionary & environmental sciences

For a reference copy of the document with all sections, see nature.com/documents/nr-reporting-summary-flat.pdf

Life sciences study design

All studies must disclose on these points even when the disclosure is negative.

Sample size	We analyzed transcriptomic and genomic data from TCGA (n=530), two microarray cohorts (A: n=865; B: n=609), the METABRIC (n=867), and the SCAN-B (n=1636) datasets.
Data exclusions	we focus on ER+/HER2- breast cancers with in silico RS <26.
Replication	N/A
Randomization	N/A
Blinding	N/A

Reporting for specific materials, systems and methods

We require information from authors about some types of materials, experimental systems and methods used in many studies. Here, indicate whether each material, system or method listed is relevant to your study. If you are not sure if a list item applies to your research, read the appropriate section before selecting a response.

Materials & experimental systems

n/a	Involved in the study
☒	☐ Antibodies
☒	☐ Eukaryotic cell lines
☒	☐ Palaeontology and archaeology
☒	☐ Animals and other organisms
☒	☐ Clinical data
☒	☐ Dual use research of concern

Methods

n/a	Involved in the study
☒	☐ ChIP-seq
☒	☐ Flow cytometry
☒	☐ MRI-based neuroimaging
